# Computational Characterization of the Binding Properties of the HIV1-Neutralizing Antibody PG16 and Design of PG16-Derived CDRH3 Peptides

**DOI:** 10.3390/biology12060824

**Published:** 2023-06-06

**Authors:** Manuel Deubler, Lucas Weißenborn, Simon Leukel, Anselm H. C. Horn, Jutta Eichler, Heinrich Sticht

**Affiliations:** 1 Division of Bioinformatics, Institute of Biochemistry, Friedrich-Alexander-Universität Erlangen-Nürnberg (FAU), 91054 Erlangen, Germany; manuel.deubler@fau.de (M.D.); anselm.horn@fau.de (A.H.C.H.); 2 Department of Chemistry and Pharmacy, Friedrich-Alexander-Universität Erlangen-Nürnberg (FAU), 91058 Erlangen, Germany; lucas.weissenborn@fau.de (L.W.); simon.leukel@fau.de (S.L.); jutta.eichler@fau.de (J.E.); 3 Erlangen National High Performance Computing Center (NHR@FAU), Friedrich-Alexander-Universität Erlangen-Nürnberg (FAU), 91058 Erlangen, Germany

**Keywords:** antibody, PG16, HIV-1, peptides, antibody mimetic peptides, molecular dynamics

## Abstract

**Simple Summary:**

Antibodies play a critical role in the immune system’s defense against pathogens. Such antibodies can be used for passive immunization, but they are rather expensive and difficult to produce. Alternatively, their structures can be used as a template for the design of peptides that retain the antibodies’ binding behaviour, but which are smaller in size and therefore more likely to be able to reach sterically shielded epitopes. However, identifying high-affinity sequences in antibody structures is challenging. In this context, we investigated the structural properties of the broadly neutralizing antibody PG16 that binds to the gp120 subunit of the HIV-1 envelope (Env) protein. Recognition occurs primarily through the complementarity determining region (CDR) H3 of PG16, which contains a tyrosine sulfation site. Molecular modeling and simulation of the protein dynamics reveal that the CDRH3 represents an energetic hotspot of PG16-gp120 recognition, and that sulfation plays an important role in enhancing the interaction. Moreover, we experimentally demonstrated that a CDRH3-derived peptide is still able to recognize gp120 with high affinity. Binding was further enhanced by the introduction of a disulfide bond that increases structural stability. These results suggest that the continued optimization of PG16-derived peptides might ultimately lead to an alternative therapeutic approach to combat HIV-1 infection.

**Abstract:**

PG16 is a broadly neutralizing antibody that binds to the gp120 subunit of the HIV-1 Env protein. The major interaction site is formed by the unusually long complementarity determining region (CDR) H3. The CDRH3 residue Tyr100H is known to represent a tyrosine sulfation site; however, this modification is not present in the experimental complex structure of PG16 with full-length HIV-1 Env. To investigate the role of sulfation for this complex, we modeled the sulfation of Tyr100H and compared the dynamics and energetics of the modified and unmodified complex by molecular dynamics simulations at the atomic level. Our results show that sulfation does not affect the overall conformation of CDRH3, but still enhances gp120 interactions both at the site of modification and for the neighboring residues. This stabilization affects not only protein–protein contacts, but also the interactions between PG16 and the gp120 glycan shield. Furthermore, we also investigated whether PG16-CDRH3 is a suitable template for the development of peptide mimetics. For a peptide spanning residues 93-105 of PG16, we obtained an experimental EC_50_ value of 3nm for the binding of gp120 to the peptide. This affinity can be enhanced by almost one order of magnitude by artificial disulfide bonding between residues 99 and 100F. In contrast, any truncation results in significantly lower affinity, suggesting that the entire peptide segment is involved in gp120 recognition. Given their high affinity, it should be possible to further optimize the PG16-derived peptides as potential inhibitors of HIV invasion.

## 1. Introduction

Human immunodeficiency virus type 1 (HIV-1), which causes acquired immuno-deficiency syndrome (AIDS) in humans, was discovered 40 years ago [1,2]. However, despite major scientific efforts, the virus remains a serious health threat worldwide. In 2021, 38.4 million people were living with HIV and 1.5 million people became newly infected. In total, 40.1 million people have died from AIDS-related illnesses since the start of the epidemic [3]. To date, there is no vaccine available to prevent HIV infection or to eradicate the virus from infected individuals. Therefore, alternative strategies are needed to combat the virus effectively and specifically.

In this context, particular attention is given to broadly neutralizing antibodies (bnAbs) that are developed over time by some HIV-infected individuals [4,5]. As part of a strategy called passive immunization, such bnAbs are recombinantly expressed and administered to other infected individuals to fight the disease. Passive immunization studies in animal models have shown that bnAbs can completely protect against viral infection [6,7,8] and are currently being tested in clinical trials [5,9]. In an alternative therapeutic approach, bnAbs serve as templates for the design of protein mimics derived from the antigen-binding site that retain the binding characteristics of the antibody [10,11,12].

**Figure 1:**
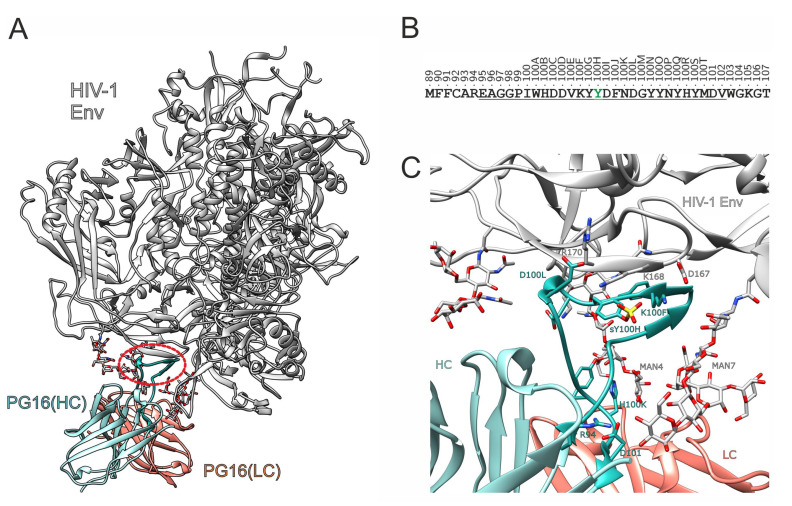
Structure of the PG16-Env complex. (**A**) Experimental structure of the PG16-Env complex (PDB code: 6ULC; [16]). The heavy and light chain of the antibody are shown in cyan and orange, respectively, and HIV-1 Env is shown in white. The red oval frames the CDRH3, which adopts the form of a hammerhead. Glycans are shown in stick presentation. (**B**) Sequence of PG16-CDRH3 (underlined) including flanking residues. The positions are labeled using the Kabat numbering scheme for antibodies [17]. Position 100H highlighted in green marks the tyrosine residue that can be post-translationally sulfated. (**C**) Enlargement of the binding site showing the interactions of CDRH3. Important residues of CDRH3 are shown as cyan sticks; interacting gp120 residues and glycans are colored according to their atom types. The sulfo group is modeled in the structure to illustrate its putative position in the interface.

BnAbs target conserved regions of the HIV-1 envelope (Env) glycoprotein, which is composed of three gp160 subunits that are processed by proteolysis to form gp120 and gp41 [13]. There are a variety of bnAbs that target different epitopes on the Env surface [4]. The antibody PG16 investigated in the present study, which neutralizes 70–80% of HIV isolates [14,15], binds to an epitope formed by the glycosylated V1–V2 region of gp120 (Figure 1A).

Specific PG16 binding is mainly achieved through the complementary determining region (CDR) number 3 of the heavy chain (CDRH3). PG16 has an exceptionally long (28 residues) CDRH3 (Figure 1B) that adopts an unusual conformation and is therefore referred to as a “hammerhead” [14]. Another peculiarity of PG16 is that the tyrosine at position 100H can be sulfated [14].

Tyrosine sulfation, a post-translational modification, is mediated by the enzyme tyrosyl-protein sulfotransferase during protein maturation in the Golgi apparatus. Through the combination of negative charge and aromatic character, sulfotyrosine (TYS) residues can increase the affinity and specificity of protein–protein interactions [18,19]. Tyrosine sulfation is also a widely used principle in HIV-1 Env recognition. Both CXCR4 and CCR5, the major chemokine receptors used as coreceptors in HIV-1 entry, contain sulfotyrosines in their N-termini [18]. In CCR5, Tys10 and Tys14 are required for proper gp120–CCR5 interaction, whereas sulfation at residues Tyr3 or Tyr15 is dispensable [18,20].

Tyrosine sulfation is also observed for several bnAbs that target gp120, e.g., PG16, PG9, E51, and 412d [18]. A quantitative analysis for the 412d antibody has shown that tyrosine sulfation at position Tyr100 or at position Tyr100C, or dual sulfation at both positions (Tyr100 and Tyr100C), leads to an increase in affinity for gp120 of 4.5-fold, 212-fold, or 500-fold, respectively [21]. For PG16, mass spectrometry revealed that the antibody can exist both in a non-sulfated and a singly sulfated form, with the latter having a higher neutralization potency [14]. Because no experimental structure of intact Env in complex with sulfated PG16 is available to date, the only structural information on the role of the sulfo group comes from a complex of sulfated PG16 with the V1-V2 fragment of gp120 [22]. For the unmodified PG16, there is a cryo-EM structure available in complex with an intact and fully glycosylated Env protein [16]. In this complex, PG16 adopts a slightly different binding mode compared to the interaction with the isolated V1-V2 fragment [16]. Therefore, the information about the interactions of the sulfo group in the truncated complex does not readily translate to the full-length complex of Env and PG16.

For a detailed analysis of the effects of PG16 sulfation, we modeled a sulfated PG16 in complex with the intact fully glycosylated Env protein (Figure 1C) and compared the dynamics and energetics with those of the unmodified complex. We calculated an energy profile for the PG16-gp120 interface in order to identify those interactions that are enhanced by sulfation. In addition, we used the information about the PG16-gp120 interaction to derive PG16 peptides that still have high gp120-binding affinity. These peptides, which were then further optimized by the introduction of disulfide bonds to increase their conformational stability, may represent an alternative approach to disrupt gp120 receptor recognition during HIV-1 infection.

## 2. Materials and Methods

### 2.1. Parameterization of Sulfotyrosine

Although sulfotyrosine (TYS) has been used previously in molecular dynamics simulations [23,24,25], parameters for the Amber parm14SB force field were not available and had to be generated for the present study. In order to ensure maximum consistency with the protein force field parameters used, ff14SB atom types were assigned to TYS. Atom types for original TYR were obtained via xleap, and atom names according to the PDB [26] were used for TYS [27].

The initial structure of the dipeptide ACE-TYS-NME was generated with Sybyl7.3 (Tripos Inc., St. Louis, MO, USA, 1991–2008). Molden [28] was used to create a Z-matrix file and set the Ramachandran angles to (−60, −40) and (−120, +120) for α- and β-conformation, respectively. For RESP charge derivation, the two backbone conformers were used. The respective sidechain conformation was chosen in a process similar to that of forcefield_PTM [29] via a simulated annealing procedure for the dipeptide with fixed backbone atoms. Gaussian16 [30] was used to subject both conformations to a geometry optimization on the HF/6-31G* level according to the original Amber charge generation procedure. In the optimized conformations, the sulfo group did not directly interact with the backbone, which would mutually influence the atomic charges. Calculation of the molecular vibrations ensured the true minimum character of the two structures. The actual RESP charge derivation was performed via RED server [31] using the two optimized α- and β-conformations as input. Peptide group atom charges were constrained to values from negatively charged GLU/ASP, in accordance with our previous parameterization approach for phosphorylated amino acids [32]. Further missing force field parameters were assigned with parmchk2 from the Amber suite [33]. Parameters are available as Supplementary Materials.

### 2.2. Generation of Starting Structures

All simulations were based on the cryo-EM structure of Fab of PG16 bound to glycosylated HIV-1 Env (PDB ID: 6ULC) [16]. The post-translational modification of residue Y100H to sulfotyrosine that is absent in this structure was achieved by first mutating Y100H to phosphotyrosine using the SwissSidechain [34] plugin for UCSF Chimera [35] and subsequently changing the phosphor atom to sulfur. To reduce the calculation time, only the variable domains of the antibody heavy and light chain were included in the simulation setup. Only glycan structures in close proximity to the CDR3 of the heavy chain (CDRH3) were kept. Specifically, these are the glycans attached to N156 and N160 of chain A and N160 of chain E. Simulations of CDRH3-derived peptides started from the backbone conformation observed for PG16 in the complex structure 6ULC. To ensure consistency with the peptides used in the experimental binding assays, these peptides were simulated with a phosphorylated Y100H instead of the sulfation present in the intact PG16.

### 2.3. Molecular Dynamics Simulations

All simulations were performed with AMBER (version 22) [33] using the ff14SB [36] for proteins and the GLYCAM06j force field [37] for glycans. For the phospho-tyrosine in the peptides, the available phosaa10 parameters [32,38] were used and the sulfotyrosine of PG16 was described by the parameters derived in this work (see Section 2.1). All systems were solvated with TIP3P water [39], neutralized with either Na^+^ or Cl^−^ ions, and placed in a truncated octahedron-shaped solvation box with a minimum distance of 15 Å between the solute and borders of the box. The simulations were performed according to a previously established protocol [40,41]. Initially, a three-step minimization was carried out to optimize the geometry of the starting structures. In the first step, all atoms except water were restrained with a harmonic potential of 5 kcal/(mol Å[b]2). In the second step, the restraints were only applied to the non-hydrogen atoms of the solute. In the last step, no restraints were applied to the whole system. Each minimization step started with 2500 steps of steepest descent algorithm followed by 2500 steps of conjugate gradient minimization. After minimization, relaxation of the systems was achieved in a short two-step MD simulation. In the first phase ( 0.1 ns), the temperature of the systems was raised from 10K–310K for the antibody–antigen systems or to 293 K for the peptide systems. During this phase, a restraint of 5 kcal/(mol Å[b]2) was applied to the protein and glycan atoms. In the second phase ( 0.4 ns), the restraint was only applied to the C_α_ atoms. This relaxation was followed by 1 μs production runs at the given temperature controlled by Berendsen thermostat without any restraints. Constant pressure periodic boundaries conditions were used at 1 bar and isotropic position scaling. The SHAKE algorithm was applied to all bonds including hydrogens [42], thus allowing a timestep of 2 fs for all MD simulations. The particle mesh Ewald method was used for computing the long-range electrostatic interactions [43]. Minimization and relaxation were performed on CPUs, while the production phase was performed on Nvidia A40 GPUs using pmemd.cuda [44,45,46]. Analysis of the trajectories was performed using cpptraj [47]. This includes RMSD, secondary structure, distance and MM/GBSA [48] using the MMPBSA.py script [49]. Graphs were plotted using Python and Biopython [50]. Structure visualizations were created using chimera [35].

### 2.4. Peptide Synthesis

Peptides (Table 1) were synthesized as C-terminal amides by Fmoc/tBu-based solid-phase synthesis on TentaGel SRAM resin, using an automated multiple peptide synthesizer, as previously described [51]. Briefly, peptides were synthesized as C-terminal amides by Fmoc/tBu-based solid-phase synthesis on TentaGel SRAM resin ( 110 mg, 0.23 mmol/g) using an automated multiple peptide synthesizer (ResPep SL by Intavis Inc., Tübingen, Germany). In peptides containing disulfides, the N-terminal cysteine residue was coupled as Fmoc-Cys(StBu)-OH. Peptides were cleaved from the resin by use of trifluoroacetic acid (TFA)/water/phenol/thioanisole/triisopropylsilane 80:5:5:5, precipitated in cold tert-butyl methyl ether, extracted with water, and lyophilized. Peptides were purified by preparative HPLC (Phenomenex Kinetex C18 column, 100 × 21.2 mm, flow rate 30 mL/min), with a gradient of acetonitrile in H_2_O (both containing 0.1% TFA, 25–60% over 10 min). Disulfide bridges were formed after a first purification step, by disulfide exchange at 0.3 mg/mL in 50% acetonitrile in ammonium carbonate (pH 8, 0.1 M) for 12 h. The reaction was monitored by analytical HPLC with ESI mass spectrometry detection (LC-MS, conditions: Phenomenex Kinetex 2.6 μm C18 100 Å column, 50 × 2.1 mm, flow rate 0.55 mL/min), with a gradient of acetonitrile in H_2_O (both containing 0.1% TFA, 5–95% over 15 min.) Oxidized peptides were again purified by preparative HPLC. Stock solutions of purified peptides were prepared at 10 mM in DMSO.

### 2.5. Peptide Binding Assays

High-binding Corning Costar microtiter half-area plates were coated with Neutravidin (Thermo Scientific, Dreieich, Germany, 4.0 μg/mL) in 0.1 M carbonate buffer pH 9.6, overnight at 4 ${}^{\circ }C$. After the blocking of unspecific binding with 1% BSA in 0.1 M phosphate buffer pH 7.2, for 1 h, the neutravidin-coated plate was incubated with 2 μm biotinylated peptide for 2 h. Plates were then incubated for 3 h with gp120 HxBc2 (ImmuneTech, New York, NY, USA) at three-fold serial dilutions, starting at 16.6 nM. Bound gp120 was detected with sheep anti-gp120 (Aalto Bio Reagents, Dublin, Ireland) 0.1 μg/mL) for 1 h, followed by rabbit anti-sheep-HRP conjugate (Jackson Immuno Research, Ely, UK, 0.1 μg/mL) for 1 h. All peptides and proteins were diluted in 0.1 M phosphate buffer pH 7.2, containing 0.01% Tween. Plates were developed with OPD ( 1 mg/mL) in the presence of 0.03% H_2_O_2_ for approximately 10 min in the dark. After the reaction was stopped with 2 M H_2_SO_4_, absorbance was read at 492 nm. The absorbance of the blanks (samples without peptide) was subtracted. All data points present means of duplicates. Curves were fitted and EC_50_ values calculated using the program Origin 2021 (OriginLab Corporation, Northampton, MA, USA).

## 3. Results and Discussion

### 3.1. Effect of Sulfation on PG16 Dynamics and Interactions

To investigate the role of tyrosine sulfation in the PG16-gp120 interaction, residue Y100H of PG16 was modified to sulfotyrosine (TYS). The sulfo group could be accommodated in the complex structure (PDB: 6ULC [16]) without any steric clashes after energy minimization (Figure 1C). 1 μs molecular dynamics (MD) simulations were performed for the unmodified PG16 (TYR-PG16) and the sulfated PG16 (TYS-PG16) in complex with HIV-1 Env.

**Figure 2:**
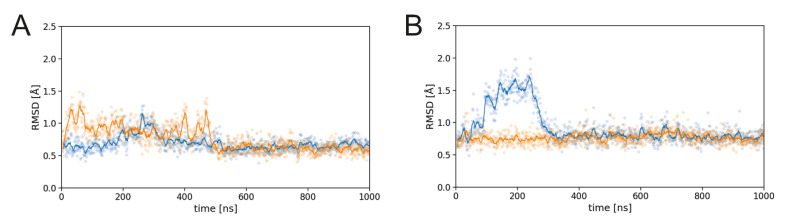
Dynamics of PG16-CDRH3 in the PG16-Env complex. RMSD for (**A**) TYS-PG16 and (**B**) TYR-PG16. Data for the first and second simulation run are shown in blue and orange, respectively. In both plots, explicit values are highlighted as dots and running averages are shown as lines.

The backbone RMSD of the CDRH3, which harbors the sulfation site Y100H, is rather similar for the TYR-PG16 and TYS-PG16 simulations (Figure 2).

**Figure 3:**
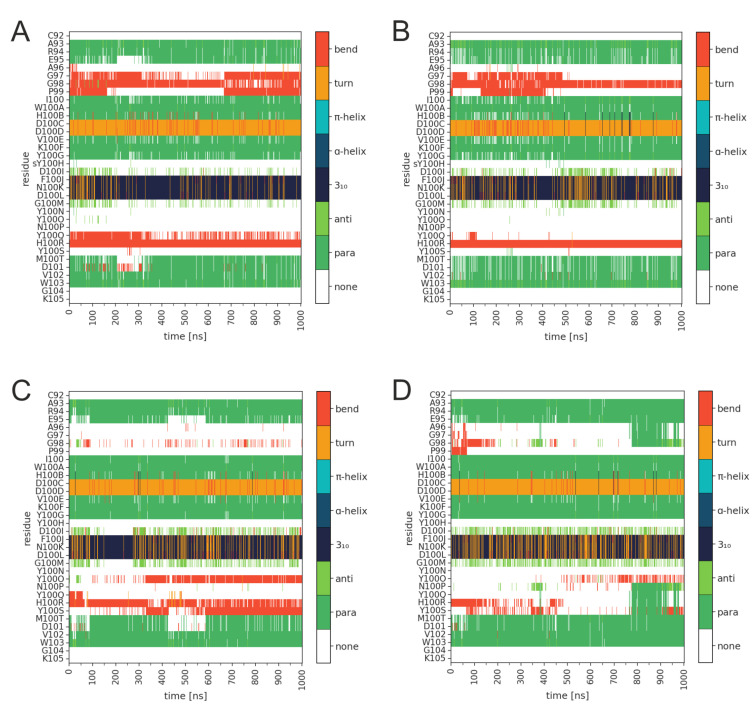
Secondary structure of the PG16-CDRH3 in the PG16-Env complex for (**A**,**B**) TYS-PG16 and (**C**,**D**) TYR-PG16. The two panels for each system show the two independent simulation runs. The color code for the different types of secondary structure is given in the bar on the right.

Apart from some transient fluctuations in the first half of the simulations, all RMSD values level off at 0.5Å–1.0Å in the second half of the simulations, indicating that the CDRH3 remains stably folded. This stable behavior is confirmed by an analysis of the secondary structure over the simulation time (Figure 3). In all simulations, the characteristic structure of the hammerhead consisting of a β-hairpin centered around a turn at position D100C/D100D is retained. Post-translational modification at position Y100H neither affects the conformation of this hairpin, nor the structure of the C-terminally adjacent structural element (classified either as a turn or a 3_10_-helix that spans F100J-D100L according to dssp analysis; Figure 3). In summary, these data show that the CDRH3 backbone secondary structure is stable for both the TYR-PG16 and TYS-PG16.

**Figure 4:**
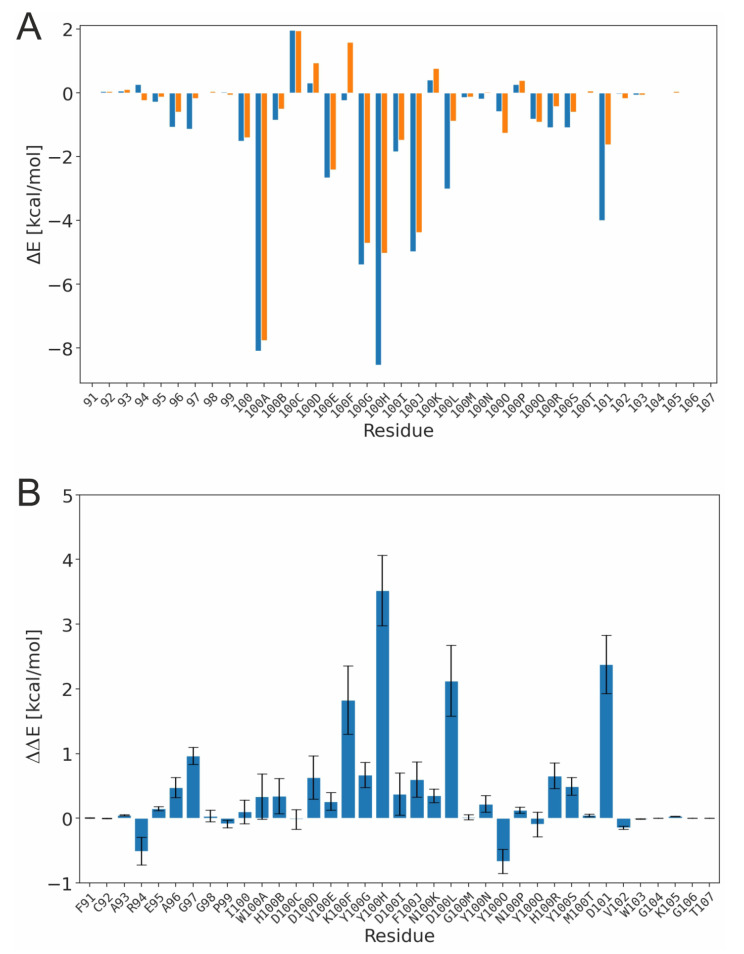
Energetics of the PG16-CDRH3 in the PG16-Env complex. (**A**) Interaction energy derived from a per-residue binding energy decomposition. Values for TYS-PG16 and TYR-PG16 are shown in blue and orange, respectively. Negative values indicate a favorable contribution to binding. Values are averaged over two simulations runs for each system. (**B**) Differences in the energetic contributions of individual residues between TYS-PG16 and TYR-PG16. Positive values indicate a stronger interaction of the respective residue in TYS-PG16. The standard error is indicated by error bars.

In the next step, we investigated the energetics of the TYR-PG16 and TYS-PG16 interaction with HIV-1 Env in more detail. A residue-level decomposition of the binding energy (Figure 4A) reveals the hotspots of the PG16-Env interaction. The largest energetic contributions come from the aromatic residues W100A, Y100G, Y100H, and F100J. These data are in line with a previous mutagenesis study of Pejchal [14], which found that the Y100G-F100J sequence stretch is crucial for the binding and neutralization of HIV-1. In addition, the same study showed that a single W100A mutation increased the IC_50_ for HIV-1 neutralization by more than 50-fold. When comparing TYR-PG16 and TYS-PG16 (Figure 4B), differences in energetic contributions are not only observed for the site of modification itself (Y100H), but also for adjacent residues such as K100F and D100L, or for the more distant D101. We have therefore inspected the interactions of these residues in more detail and compared them between the unmodified and the sulfated PG16.

**Figure 5:**
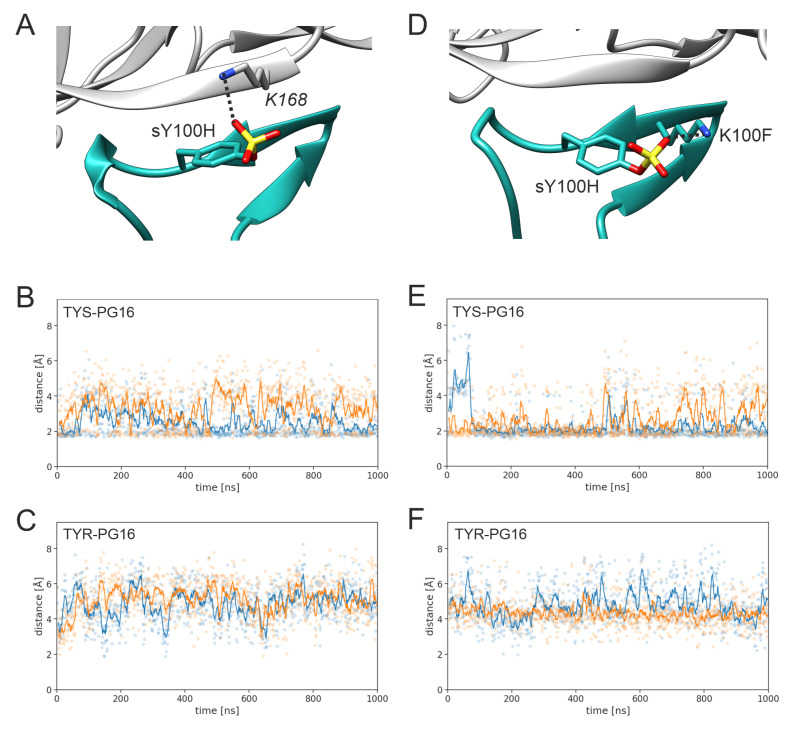
Key interactions of PG16 residue 100H. (**A**) Intermolecular interaction of sY100H with K168 of gp120. (**B**) Shortest distance between the sY100H sulfo group and the K168 ammonium group (run1, blue; run2, orange). Explicit values are highlighted by dots and running averages are shown as lines. (**C**) Shortest distance between the Y100H sidechain hydroxyl group and the K168 ammonium group. (**D**) Intramolecular interaction of sY100H with K100F of PG16. (**E**) Shortest distance between the sY100H sulfo group and the K100F ammonium group (run1, blue; run2, orange). (**F**) Shortest distance between the Y100H sidechain hydroxyl group and the K100F ammonium group.

The sulfate group of sY100H forms polar interactions with K168 of gp120 (Figure 5A). This interaction persists over the entire simulation time, although some transient fluctuations in the distance are observed (Figure 5B). In contrast, the Y100H-K168 distance is considerably larger for the unmodified tyrosine (Figure 5C). Although no final conclusion can be drawn on the basis of two simulations for each system, our data show a trend toward more stable interactions in the sulfated system. The absence of the sulfo group not only increases the distance of the two side chains, but also reduces electrostatic complementarity due to the lack of a negative charge. Together, these two factors explain the significant energetic benefit of sulfation at this position (~3 kcal/mol; Figure 4B). This role of Y100H sulfation to increase electrostatic complementarity is also in line with the previous study of Pancera et al. [22], who found that the sulfo group forms an intermolecular salt bridge in a complex between PG16 and the V1-V2 fragment of gp120. In addition to this intermolecular interaction, sY100H also forms an intramolecular interaction with K100F (Figure 5D). This interaction is rather stable over the simulation time in TYS-PG16 (Figure 5E) but cannot be formed by the shorter unmodified sidechain in TYR-PG16 (Figure 5F).

**Figure 6:**
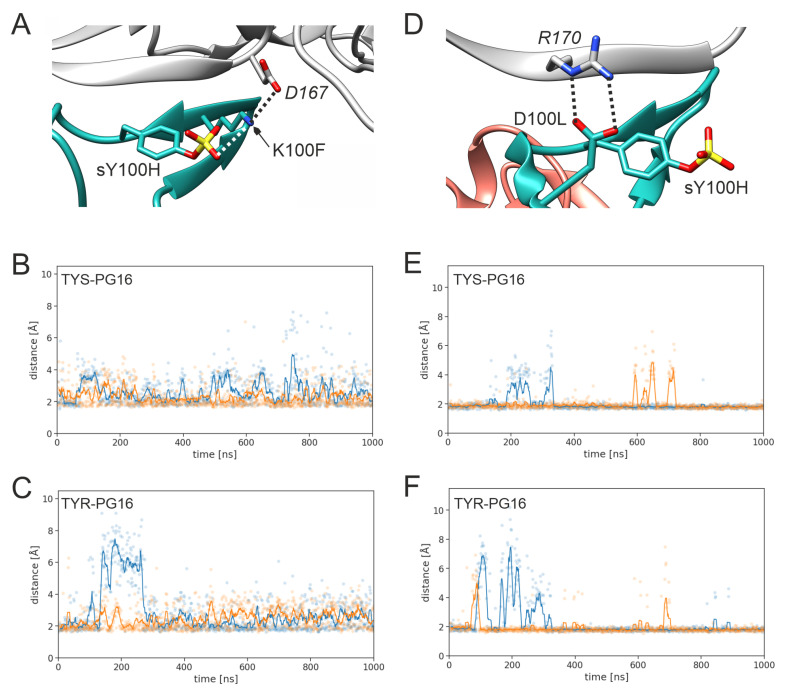
Key interactions of PG16 residues K100F and D100L. (**A**) Intermolecular interaction of K100F with D167 of gp120 (black dotted line). In addition, the intramolecular sY100H-K100F interaction is indicated by a white dotted line. (**B**,**C**) Shortest distance between the K100F ammonium group and the D167 carboxy group in (**B**) TYS-PG16 and (**C**) TYR-PG16 (run1, blue; run2, orange). Explicit values are highlighted as dots and running averages are shown as lines. (**D**) Interaction of D100L with R170 of gp120. (**C**,**D**) Shortest distance between the D100L carboxy group and the R170 guanidino group in (**E**) TYS-PG16 and (**F**) TYR-PG16 (run1, blue; run2, orange).

The intramolecular interaction that stabilizes the relative positions of sY100H and K100F also contributes to the stabilization of the intermolecular interactions of K100F. K100F forms a salt bridge with D167 of gp120 (Figure 6A). This salt-bridge is present in TYS-PG16 (Figure 6B) and TYR-PG16 (Figure 6C); however, larger distance fluctuations (>5 Å) are only observed for TYR-PG16 (Figure 6C), indicating that the network of polar interactions is less stable in unmodified PG16. A similar trend is observed for the salt bridge formed between D100L and R170 of gp120 (Figure 6D). Again, larger distances (>5 Å) are observed only for TYR-PG16 (Figure 6F) but not for TYS-PG16 (Figure 6E).

**Figure 7:**
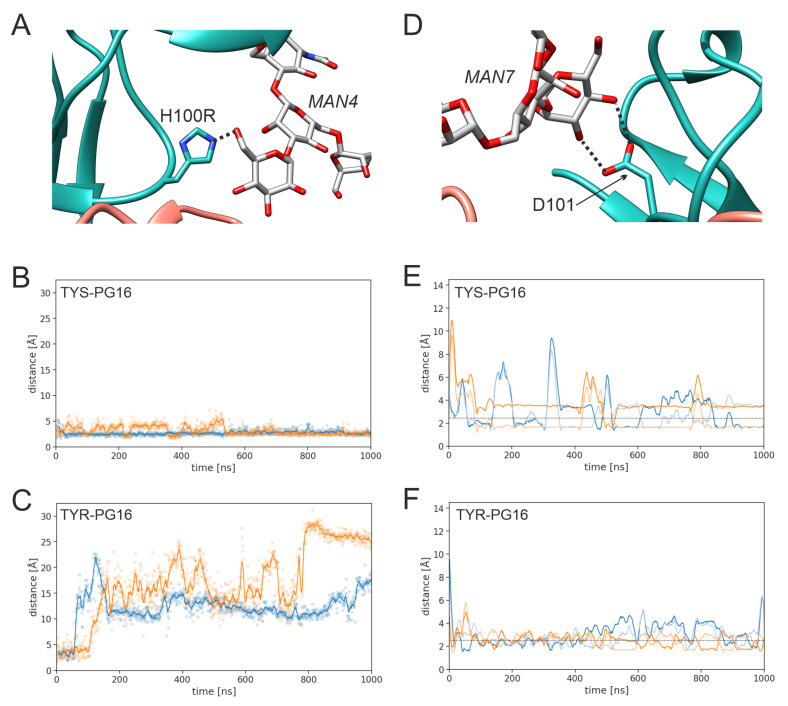
Key interactions of PG16 residues H100R and D101. (**A**) Interaction of H100R with the MAN4 glycan of gp120 (black dotted line). (**B**,**C**) Shortest distance between the H100R imidazole ring and the MAN4 glycan in (**B**) TYS-PG16 and (**C**) TYR-PG16 (run1, blue; run2, orange). Explicit values are highlighted as dots and running averages are shown as lines. (**D**) Interaction of D101 with the MAN7 glycan of gp120. (**E**,**F**) Shortest distance between the D100 carboxyl oxygens and the hydroxy groups of the MAN7 glycan (**E**) TYS-PG16 and (**F**) TYR-PG16 (run1, blue; run2, orange). The two shades of blue and orange indicate the interactions of the individual oxygens, Oδ1 and Oδ2. The black dotted line marks a distance of 2.5Å, as the upper limit for the formation of a hydrogen bond (measured between the oxygen atom as the donor and hydrogen atom as the hydrogen bond acceptor).

The enhanced stability upon sulfation also affects the interactions between PG16 and the glycans of gp120 (Figure 7). One example is H100R, which interacts with the MAN4 glycan (Figure 7A). This interaction is stable for TYS-PG16 throughout the entire simulation (Figure 7B), whereas it is lost within the first 100 ns in both simulation runs of TYR-PG16 (Figure 7C). For D101, which interacts with the MAN7 glycan (Figure 7D), the interpretation is more complicated. For TYS-PG16, this interaction is completely lost for short (< 100 ns) periods of the simulation time, while at least one hydrogen bond is formed between the D101 side chain and a hydroxyl group of MAN7 in the remaining parts of the simulation, as indicated by a distance of <2.5 Å (Figure 7E). The corresponding hydrogen bond is very stable and only marginal distance fluctuations are observed in the respective time windows (e.g., from 150 ns to 400 ns, 500 ns to 750 ns, and 850 ns to 1000 ns for run2; light orange line in Figure 7E). In contrast, in the TYR-PG16 simulation (Figure 6F), the magnitude of the fluctuations in the D101-MAN7 distance is smaller; however, short distances, indicative of stable hydrogen bonding, are observed less frequently than for PG16-TYS. For PG16-TYR, the distance mostly fluctuates around the threshold of 2.5 Å, which is considered as the upper limit for a hydrogen bond (considering the hydrogen-acceptor distance; [52]). The difference in the strength of this hydrogen bond may contribute to the stronger interaction of D101 in the TYS-PG16 system.

In summary, the data above show the trend that PG16 sulfation not only enhances gp120-binding at the site of the modification, but also has a favorable influence on polar interactions in its vicinity. In the case of K100F, this is a rather direct influence by stabilizing the K100F position, which facilitates interaction with D167. In case of the remaining interactions investigated here, the effect is more indirect, i.e., the stabilization of the interface at the site of sulfation lowers fluctuations of other intermolecular interactions. The importance of tyrosine sulfation for enhancing binding affinity has been described previously for a number or protein–protein interactions [18]. The present analysis confirms this trend and gives additional insight into the atomic details underlying this effect. The analysis performed here shows a general trend, which, however, cannot readily be quantified. Depending on the HIV-1 isolate, Env proteins differ significantly in protein sequence [53], glycation sites [54] and glycan composition [55], making quantitative statements difficult. However, PG16 is a broadly neutralizing Ab, efficient against 70% to 80% of all HIV-1 isolates [14,15], suggesting that the overall principles of the recognition and effect of sulfation are similar for different HIV-1 isolates.

### 3.2. Design of PG16-Derived Peptides

**Figure 8:**
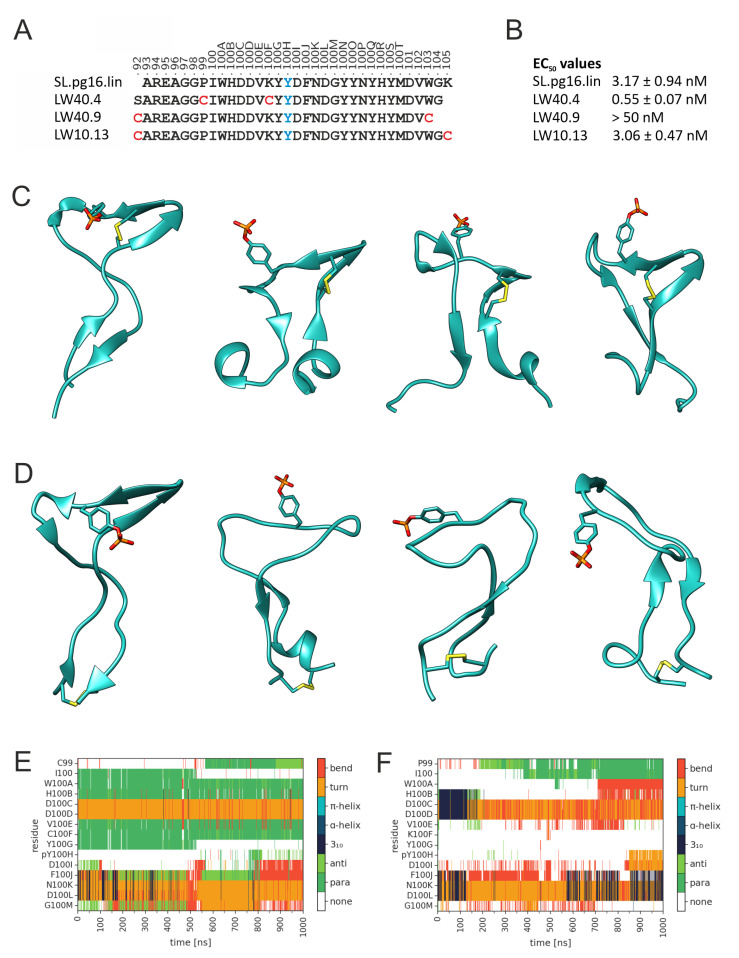
Effect of disulfide bonds on the stability of PG16-CDRH3-derived peptides. (**A**) Schematic presentation of the alternative disulfide bonding patterns investigated. Cysteines forming an intramolecular disulfide bond are highlighted in red. Position 100H highlighted in blue represents a phosphotyrosine. (**B**) Experimental EC_50_ values for binding of HIV-1 gp120 (HxBc2) to the disulfide-bonded peptides in comparison to the linear peptide SL.pg16.lin. (**C**) Snapshots from the MD simulation illustrating the dynamics of the free LW40.04 peptide. The initial structure is shown in the left panel. The position of the disulfide bond and of the phosphotyrosine is indicated as sticks. (**D**) Snapshots from the MD simulation illustrating the dynamics of the free LW40.9 peptide. The initial structure is shown in the left panel. The position of the disulfide bond and of the phosphotyrosine is indicated as sticks. (**E**,**F**) Conformational stability of the central β-hairpin in (**E**) LW40.4 and (**F**) LW40.9. The color code for the different types of secondary structure is given in the bar on the right.

The investigation above suggested that the CDRH3 of the sulfated PG16 has favorable gp120 binding properties. This prompted us to investigate whether peptides presenting CDRH3 would retain the binding properties of PG16. We initially generated a 33-residue peptide (SL.pg16.lin; Figure 8A) that covers all residues with energetically favorable contributions to the PG16-gp120 interaction (Figure 4A). Since the sulfo group was found to be hydrolysis-sensitive during peptide synthesis, phosphotyrosine (pY100H) was used as a surrogate, because it exhibits physico-chemical properties similar to those of sulfotyrosine (see Table 1 for a summary of all peptide sequences investigated). Recombinant, soluble HIV-1 gp120 (HxBc2) was found to bind with an EC_50_ of 3.17 nM to linear SL.pg16.lin (Figure 8B), making it a promising candidate for further optimization. Several previous studies have used artificial disulfide bonds to enhance the binding properties of peptides [51,56]. We also used this concept for our PG16 CDRH3 peptides by introducing disulfide bonds at different sites (Figure 8A). Initially, two different disulfide bonding patterns were investigated. In LW40.4, residues P99 and K100F were replaced by cysteine to allow for the formation of a disulfide bond that fixes the CDRH3 β-hairpin. In LW40.9, the native C92 was retained, and a second cysteine was introduced at position 103 to allow for the formation of a disulfide bond that fixes the proximity between termini of the peptide. The experimentally determined EC_50_ values (Figure 8B) show that gp120 binds with higher affinity to LW40.4 than to SL.pg16.lin, whereas the disulfide bonding pattern in LW40.9 leads to a significant decrease in affinity.

To understand the structural basis of these results, MD simulations of the free LW40.4 and LW40.9 peptides were performed. Snapshots of representative structures from the LW40.4 simulation (Figure 8C) show that the disulfide bond efficiently fixes the central β-hairpin, whereas the termini are rather flexible and deviate significantly from the conformation present in intact PG16. In contrast, the terminal disulfide bond in LW40.9 (Figure 8D) is insufficient to stabilize the central β-hairpin. The differences in β-hairpin stability between LW40.4 and LW40.9 also become evident from a plot of the secondary structure over simulation time (Figure 8E,F).

A closer inspection of the termini in LW40.9 (Figure 8D) reveals that they remain close, as expected from the presence of the disulfide bond; however, their conformation becomes significantly distorted over simulation time. For example, transient helical turns are formed near the disulfide bond (Figure 8D), which are not present in the initial structure. This finding suggests that the properties of LW40.9 might be improved by changing the position of the terminal disulfide bond. Consequently, we designed LW10.13, in which the sequence position of the C-terminal cysteine is shifted by two residues compared to LW40.9 (Figure 8A). This change leads to a significantly improved EC_50_ of LW10.13 compared to LW40.9 (Figure 8B), demonstrating that the exact position of the disulfide bond critically affects binding affinity. However, even the optimized terminal disulfide bond in LW10.13 does not result in an improved EC_50_ compared to the SL.pg.16.lin precursor (Figure 8B). This observation suggests that the stabilization of the central β-hairpin is more efficient for improving binding properties than fixing the spatial proximity of the CDRH3 termini. The role of intramolecular interactions for the maintenance of the PG16 CDRH3 structure has been previously shown in a study by Kondo et al. [57]. The authors found that a hydrogen bond between Y100Q and P99 is critical for the structural rigidity of the CDRH3. Position 99 is also used for stabilization by a disulfide bond in LW40.4, suggesting that stabilization at this site might be beneficial for CDRH3 binding properties.

In addition to the stability of the central β-hairpin, the LW40.4 simulations also revealed a high flexibility of the peptide termini (Figure 8C). In intact PG16, the residues near the termini show favorable energy contributions to the PG16-gp120 interaction (Figure 4A), which raises the question of whether this role is still retained in LW40.4 despite the high flexibility of the free peptide.

**Figure 9:**
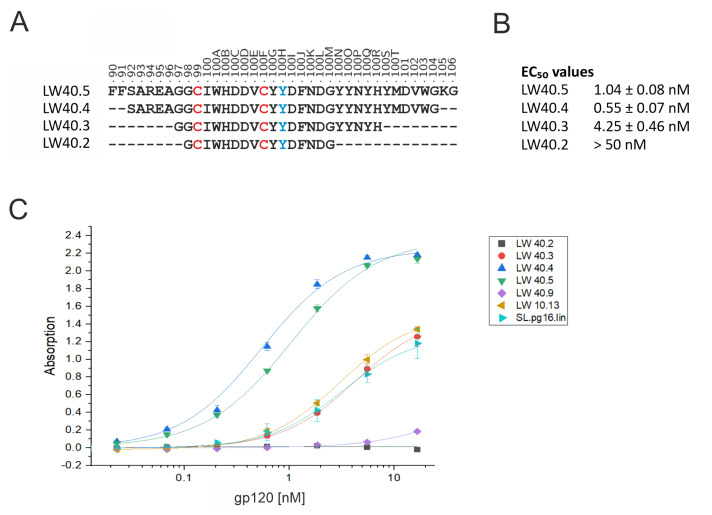
Effect of peptide length and disulfide bridges on the binding properties of PG16−CDRH3−derived peptides. (**A**) Sequence of the four peptides LW40.5, LW40.4, LW40.3, and LW40.2, which differ in their length of the N- and C-termini. Cysteines forming an intramolecular disulfide bond are highlighted in red. Position 100H highlighted in blue represents a phosphotyrosine. (**B**) Experimental EC_50_ values for binding of HIV-1 gp120 (HxBc2) to the peptides of different length. (**C**) Dose-dependent binding of gp120 binding to various PG16-derived peptides (see Table 1 for a complete list of peptide sequences). See Materials and Methods for details (Section 2.4).

To address this question, two terminally truncated versions of LW40.4 (termed LW40.3 and LW40.2) were synthesized (Figure 9A). The experimental binding data show that even a moderate truncation (LW40.3) causes a decrease in the EC_50_ by almost one order of magnitude, whereas the even shorter LW40.2 shows no detectable binding at all (Figure 9B). This result demonstrates that those residues, which were mapped as energetically important for PG16 binding (Figure 4A), are also required for the high-affinity binding of LW40.4. This finding also indicates that the binding mode of LW40.4 is similar to that of PG16. In contrast, an N- and C-terminal extension (peptide LW40.5) did not result in an enhanced EC_50_ compared to LW40.4 (Figure 9A,B) indicating that LW40.4 already contains all CDRH3 residues important for gp120 binding. For all peptides investigated in this study, the dose-dependent binding of gp120 is shown in Figure 9C. This indicates that LW40.4 exhibits the most favorable binding properties. This peptide thus represents a promising starting point for further optimization to eventually obtain a potent inhibitor that efficiently blocks HIV-1 infection.

Further optimization may include the incorporation of non-natural amino acids, D-amino acids, or the methylation of amide groups to improve proteolytic stability and/or binding affinity. In addition, LW40.4 may be covalently linked to another CDR-derived peptide that binds to an adjacent site of gp120 to create bispecific ligands.

It should be stressed here that the approach of the present study is not limited to antibodies inhibiting HIV-1 infection, but can also be applied to other infectious diseases, for which structural information on antibody–antigen complexes is available. This is, for example, the case for the interaction between antibodies and the spike protein of SARS-CoV-2, which plays a central role in viral infectivity and transmissibility [58]. Therefore, the design of inhibitory peptides derived from antibodies may represent a promising alternative therapeutic option for the treatment of infectious diseases.

## 4. Conclusions

We investigated the broadly neutralizing antibody PG16, which binds to the gp120 subunit of the HIV-1 Env protein. Our data show that the long CDRH3 represents a major site of interaction and that tyrosine sulfation at position 100H enhances binding. Although sulfation does not affect the overall conformation of the CDRH3, it strengthens the interactions both at the site of modification and for flanking residues. This stabilization includes not only protein–protein contacts, but also the interactions between PG16 and the gp120 glycan shield.

We also demonstrated that CDRH3-derived peptides retain the ability to recognize gp120 with high affinity. Depending on their sequence position, disulfide bonds proved to have either a positive or a negative effect on binding affinity. This indicates that the positions of disulfide bonds need to be carefully chosen. In contrast, peptide truncation always resulted in significantly lower affinity, suggesting that the overall binding mode of the peptides is similar to that of CDRH3 in intact PG16, where the entire CDRH3 is involved in binding. The highest-affinity peptide obtained from the present study (LW40.4) exhibits an EC_50_ of 0.55 nM for the binding of gp120 to the peptide. In future, the properties of LW40.4 may be further optimized, e.g., by the incorporation of non-natural amino acids, D-amino acids, or the methylation of amide groups, so that it can eventually be used in an anti-infective therapy.

## Figures and Tables

**Table 1:** Sequences of the peptides investigated in the present study.

Peptide	Sequence
SL.pg16.lin	Bio ^a^-Aoa ^b^-AREAGGPIWHDDVKY(pY) ^c^DFNDGYYNYHYMDVWGK-NH_2_
LW10.13	Bio-Aoa-[CAREAGGPIWHDDVKY(pY)DFNDGYYNYHYMDVWGC]-NH_2_
LW40.02	Bio-Aoa-G-[CIWHDDVC]-Y(pY)DFNDG-NH_2_
LW40.03	Bio-Aoa-GG-[CIWHDDVC]-Y(pY)DFNDGYYNYH-NH_2_
LW40.04	Bio-Aoa-SAREAGG-[CIWHDDVC]-Y(pY)DFNDGYYNYHYMDVWG-NH_2_
LW40.05	Bio-Aoa-FFSAREAGG-[CIWHDDVC]-Y(pY)DFNDGYYNYHYMDVWGKG-NH_2_
LW40.09	Bio-Aoa-[CAREAGGPIWHDDVKY(pY)DFNDGYYNYHYMDVC]-NH_2_

^a^ Bio, biotin. ^b^ Aoa, 8-amino-3,6-dioxa-octanoic acid. ^c^ (pY), phosphotyrosine. Square brackets denote disulfide bridges between cysteine residues.

## Data Availability

The force field parameters for sulfotyrosine that were generated in the present study are available as Supplementary Information.

## Supplementary Materials

The following supporting information can be downloaded at https://www.mdpi.com/article/10.3390/biology12060824/s1: ff14SB force field parameter files for sulfotyrosine (mid-chain and terminal species) including an Amber sample input.
